# Anthocyanins Inhibits Oxidative Injury in Human Retinal Pigment Epithelial ARPE-19 Cells via Activating Heme Oxygenase-1

**DOI:** 10.4014/jmb.2310.10011

**Published:** 2023-11-13

**Authors:** Cheol Park, Hyun Hwangbo, Sung Ok Kim, Jeong Sook Noh, Shin-Hyung Park, Su Hyun Hong, Sang Hoon Hong, Gi-Young Kim, Yung Hyun Choi

**Affiliations:** 1 Division of Basic Sciences, College of Liberal Studies, Dong-eui University, Busan 47340, Republic of Korea; 2 Anti-Aging Research Center, Dong-eui University, Busan 47340, Republic of Korea; 3 Department of Biochemistry, Dong-eui University College of Korean Medicine, Busan 47227, Republic of Korea; 4 Department of Food Science and Biotechnology, College of Engineering, Kyungsung University, Busan 48434, Republic of Korea; 5 Department of Food Science & Nutrition, Tongmyong University, Busan 48520, Republic of Korea; 6 Department of Pathology, Dong-eui University College of Korean Medicine, Busan 47227, Republic of Korea; 7 Department of Internal Medicine, Dong-eui University College of Korean Medicine, Busan 47227, Republic of Korea; 8 Laboratory of Immunobiology, Department of Marine Life Sciences, Jeju National University, Jeju 63243, Republic of Korea

**Keywords:** Anthocyanins, genotoxicity, apoptosis, ROS, Nrf2/HO-1

## Abstract

Anthocyanins belong to phenolic pigments and are known to have various pharmacological activities. This study aimed to investigate whether anthocyanins could inhibit hydrogen peroxide (H_2_O_2_)-induced oxidative damage in human retinal pigment epithelial ARPE-19 cells. Our results indicated that anthocyanins suppressed H_2_O_2_-induced genotoxicity, while inhibiting reactive oxygen species (ROS) production and preserving diminished glutathione. Anthocyanins also suppressed H_2_O_2_-induced apoptosis by reversing the Bcl-2/Bax ratio and inhibiting caspase-3 activation. Additionally, anthocyanins attenuated the release of cytochrome c into the cytosol, which was achieved by interfering with mitochondrial membrane disruption. Moreover, anthocyanins increased the expression of heme oxygenase-1 (HO-1) as well as its activity, which was correlated with the phosphorylation and nuclear translocation of nuclear factor-erythroid-2 related factor 2 (Nrf2). However, the cytoprotective and anti-apoptotic effects of anthocyanins were significantly attenuated by the HO-1 inhibitor, demonstrating that anthocyanins promoted Nrf2-induced HO-1 activity to prevent ARPE-19 cells from oxidative stress. Therefore, our findings suggest that anthocyanins, as Nrf2 activators, have potent ROS scavenging activity and may have the potential to protect ocular injury caused by oxidative stress.

## Introduction

Anthocyanins, water-soluble pigments, are classified into the polyphenolic flavanol family composed of anthocyanidin aglycone and one or more glycosides [1, 2]. To date, more than 700 anthocyanins have been identified in various plants, including berries, and are reported to have diverse pharmacological properties, such as anti-inflammatory, antidiabetic, antithrombotic and antiallergic effects [3-5]. In particular, accumulating studies have shown that the improvement of aging-related chronic diseases such as neurodegenerative, cardiovascular, and ocular diseases by anthocyanins is closely associated with their potent antioxidant action [3, 6, 7]. The antioxidant potency of anthocyanins is primarily related to increased reactive oxygen species (ROS) scavenging capacity through regulation of antioxidant-related signaling pathways [8-10]. For example, the alleviation of insulin resistance, inflammation and tissue damage by anthocyanins isolated from *Aronia melanocarpa* (Michx.) Ell. in type 2 diabetic mice was related to ROS-mediated downregulation of nuclear factor kappa B signaling [11]. Wang, *et al*. [12] reported that the anti-apoptotic effect of anthocyanins extracted from black bean seeds in radiation-exposed hippocampal neurons was due to the suppression of ROS generation following activation of Sirtuin-3. In addition, anthocyanins isolated from *Hibiscus syriacus* L. (Haeoreum) inhibited apoptosis in HaCaT human keratinocytes exposed to H_2_O_2_ through nuclear factor-erythrocyte 2-associated factor 2 (Nrf2)-dependent activation of heme oxygenase-1 (HO-1) [13]. The important roles of Nrf2 as an antioxidant regulator of anthocyanins have also been confirmed in models of diabetes, retinopathy and Alzheimer's disease [14-17].

Among the transcription factors involved in the antioxidant signaling pathway, Nrf2 is critically involved as a transcription factor for several cytoprotective antioxidant phase II enzymes to counteract oxidative stress [18, 19]. Moreover, HO-1, a key downstream gene of Nrf2, contributes to the regulation of redox homeostasis through its metabolites. For this reason, the Nrf2/HO-1 axis has been widely recognized as a key defense system against oxidative stress [18, 20]. Recently, it has been shown that anthocyanins can exert antioxidant effects through activation of Nrf2 in retinal pigment epithelial (RPE) cells [21, 22], but the role of this transcription factor is still limitedly known. According our previous studies, anthocyanins derived from *Vitis coignetiae* Pulliat, a type of berry rich in anthocyanins, have been demonstrated to have anticancer and anti-obesity activities through the regulation of various intracellular signal transduction pathways, but no study on their antioxidant activity has been performed [23-27]. Therefore, our study aimed to evaluate the involvement of Nrf2/HO-1 signaling pathway in the antioxidant capacity of anthocyanins isolated from *V. coignetiae* Pulliat fruits in a human RPE ARPE-19 cell line.

## Materials and Methods

### Cell Culture and Treatment

Immortalized ARPE-19 cells (ATCC, USA) were cultured in Dulbecco’s Modified Eagle’s Medium/F-12 medium [28]. Stock solutions of H_2_O_2_ (Sigma-Aldrich, USA) and anthocyanins extracted from *V. coignetiae* fruits prepared using dimethyl sulfoxide (Sigma-Aldrich) were diluted to appropriate concentrations in culture medium and then treated with cells. To examine the suppressive potential of anthocyanins on oxidative damage following H_2_O_2_ stimulation, cells were treated with anthocyanins and H_2_O_2_ for 24 h or treated with anthocyanins or/and zinc protoporphyrin IX (ZnPP, Sigma-Aldrich) for 1 h prior to stimulation with H_2_O_2_ for 24 h. To examine the protective potential of anthocyanins on ROS production by H_2_O_2_, cells were maintained in medium containing anthocyanins or/and ZnPP for 1 h before exposure to H_2_O_2_ for 1 h.

### Analysis of Cytotoxicity and Observation of Cell Morphology

Cell viability of cells exposed to anthocyanins and H_2_O_2_ alone or stimulated with H_2_O_2_ in the presence of anthocyanins or/and ZnPP was examined using the 3-(4,5-dimethylthiazol-2-yl)-2,5-diphenyltetrazolium bromide (MTT) assay [29]. At the same time, images of morphological changes in cells were captured using an optical microscope (Carl Zeiss, Germany).

### Comet Assay

To assess DNA damage, the Comet Assay Kit purchased from Trevigen, Inc. (USA) was used according to the manufacturer’s protocol. In brief, cells stimulated with H_2_O_2_ with or without anthocyanins were mixed in 1%agarose, spread evenly on a slide, and subjected to DNA denaturation and electrophoresis. Subsequently, images were visualized using fluorescence microscopy (Carl Zeiss) for each treatment group after staining with an asymmetric cyanine dye.

### 8-Hydroxyguanosine (8-OHdG) Assay

Levels of 8-OHdG, an RNA nucleoside that is an oxidized derivative of guanosine, were quantified using the 8-OHdG Assay Kit (Abcam, Inc., UK) following to the manufacturer's instructions. The absorbance of each treatment group was determined at 450 nm, and results were presented as ng of 8-OHdG/ml.

### Western Blot Analysis

Total proteins were isolated according to previously described methods [30]. The mitochondrial, nuclear and cytoplasmic proteins were prepared using a Mitochondrial Fractionation Kit (Thermo Fisher Scientific, USA) or Cytoplasmic and Nuclear Protein Extraction Kit (Sigma-Aldrich). After protein quantification, Western blot analysis was performed using an equal amount of protein. After protein quantification, Western blot analysis was performed using the same amount of protein, antibodies to be analyzed, and SuperSignal West Pico PLUS (Thermo Fisher Scientific) [30]. Expression of actin, lamin B, and cytochrome c oxidase IV (COX IV) was presented as housekeeping proteins for total, nuclear, and mitochondrial proteins, respectively. Antibodies used for this study were purchased from Thermo Fisher Scientific, Cell Signaling Technology (USA), Abcam, Inc., and Santa Cruz Biotechnology, Inc. (USA) (Table 1).

### Apoptosis and Mitochondrial Membrane Potential (MMP) Analysis

The degree of apoptosis was analyzed using the Annexin V-FITC Apoptosis Staining/Detection Kit (Abcam, Inc.). In brief, the collected cells were stained with annexin V/propidium iodide (PI), and annexin V-positive cell populations were regarded as apoptosis-induced cells using flow cytometry. To evaluate MMP levels, cells were stained with 5,5',6,6'-tetrachloro-1,1',3,3'-tetraethylbenzimidazolylcarbocyanine iodide (JC-1, Thermo Fisher Scientific). The frequency of cells lacking MMP was expressed as a percentage of JC-1 monomer.

### Nuclear Morphology Analysis

To monitor apoptosis using 4',6-diamidino-2-phenylindole (DAPI) dye, the nuclei were stained with DAPI solution (Sigma-Aldrich) according to previous methods [31]. Subsequently, images of DAPI-stained nuclei were acquired under a fluorescence microscope.

### Caspase-3 Activity Assay

Differences in the enzymatic activity of caspase-3 in each treatment group were evaluated using a commercially available kit (Thermo Fisher Scientific). Cell lysates, lysed using reagents provided by the manufacturer, were read at 485/530 nm using a microplate reader, according to the manufacturer's instructions. Caspase-3 activity in each treatment group is presented compared to that in the control group.

### ROS Detection

For quantitative evaluation of total intracellular ROS levels, 2',7'-dichlorofluorescein diacetate (DCF-DA) dye was used. At the end of the incubation time, the harvested cells were reacted with the DCF-DA solution (Sigma-Aldrich) and the fluorescence signal of DCF, indicating ROS production, was immediately measured using flow cytometry [32]. Fluorescence imaging was also conducted with a fluorescence microscope to detect differences in emitted DCF fluorescence intensity.

### Measurement of Glutathione/Glutathione Disulfide (GSH/GSSG) Ratio

Alterations in the GSH/GSSG ratio for each treatment group were quantified using a GSH/GSSG Analysis Kit obtained from Abcam, Inc. Briefly, cells of each treatment group were reacted under the conditions recommended by the manufacturer, and then the concentrations of GSH and GSSG were calculated based on the standard curve of reduced GSH and oxidized GSSG.

### HO-1 Activity Assay

To detect HO-1 activity, bilirubin concentrations were assessed using the HO-1 ELISA kit purchased from Abcam, Inc. In brief, bilirubin levels in each treatment group were quantified based on absorbance at 510 nm, according to the manufacturer’s method.

### Statistical Analysis

The results of each experiment were presented as mean ± standard deviation (SD). Statistical significance was performed using GraphPad Prism and set at *p* < 0.05 (**p < 0.01 and ***p < 0.001 compared to the control group; ^#^*p* < 0.05 and ^###^*p* < 0.001 compared to H_2_O_2_-treated cells; ^$$$^*p* < 0.001 compared to anthocyanins + H_2_O_2_ treatment group).

## Results

### Anthocyanins Abolished H_2_O_2_-Induced Decrease in ARPE-19 Cell Viability

**Fig. 1:**
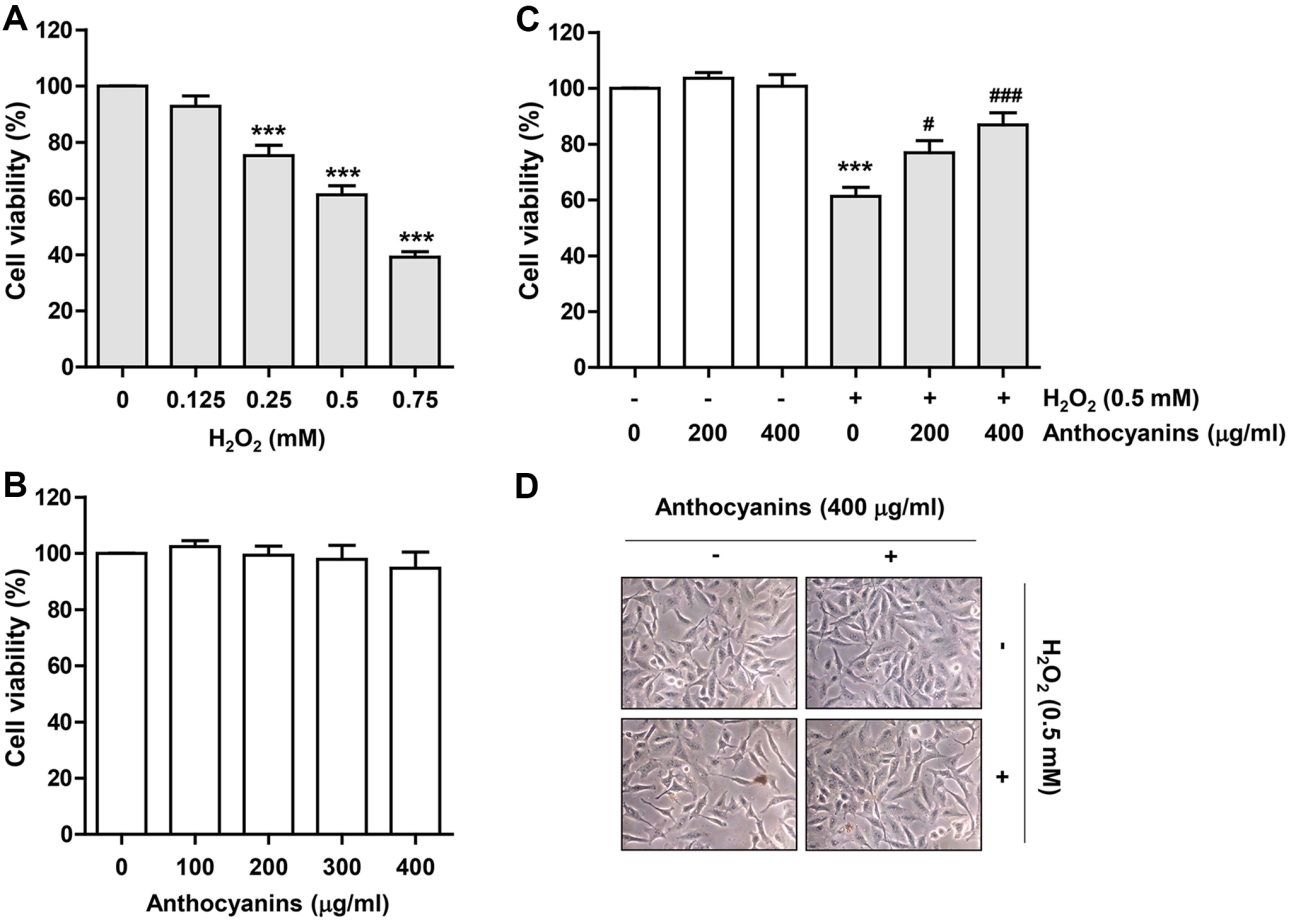
Anthocyanins protected H_2_O_2_-induced reduction of cell viability in ARPE-19 cells. (**A-C**) Results of MTT assay analyzed after treating cells with different concentrations of H_2_O_2_ (**A**) or anthocyanins (**B**) for 24 h or pre-treating cells with anthocyanins for 1 h and then treating them with H_2_O_2_ for 24 h (**C**). (**D**) Representative morphological images of cells cultured under different conditions (200×).

To examine the inhibitory potential of anthocyanins against H_2_O_2_-induced cytotoxicity in ARPE-19 cells, cell viability was determined using MTT assay. As shown in Fig. 1A, cell viability was suppressed in a dose-dependent manner in cells after H_2_O_2_ exposure, and the cell viability of ARPE-19 cells cultured in medium containing 0.5 mM H_2_O_2_ was suppressed by about 60%. Therefore, in all subsequent experiments, 0.5 mM was selected as the concentration of H_2_O_2_ treatment to mimic oxidative damage. And since anthocyanins did not induce significant inhibition of cell viability at treatment concentrations within the maximum 400 μg/ml, 400 μg/ml was set as the highest and optimal concentration (Fig. 1B). And as a result of evaluating the inhibitory effect of H_2_O_2_-mediated cytotoxicity of anthocyanins, it was found that pretreatment with anthocyanins at 400 μg/ml significantly raised cell viability up to about 86% and inhibited morphological changes of shrunken and thinned cells (Fig. 1C and 1D).

### Anthocyanins Protected DNA Damage in H_2_O_2_-Stimulated ARPE-19 Cells

**Fig. 2:**
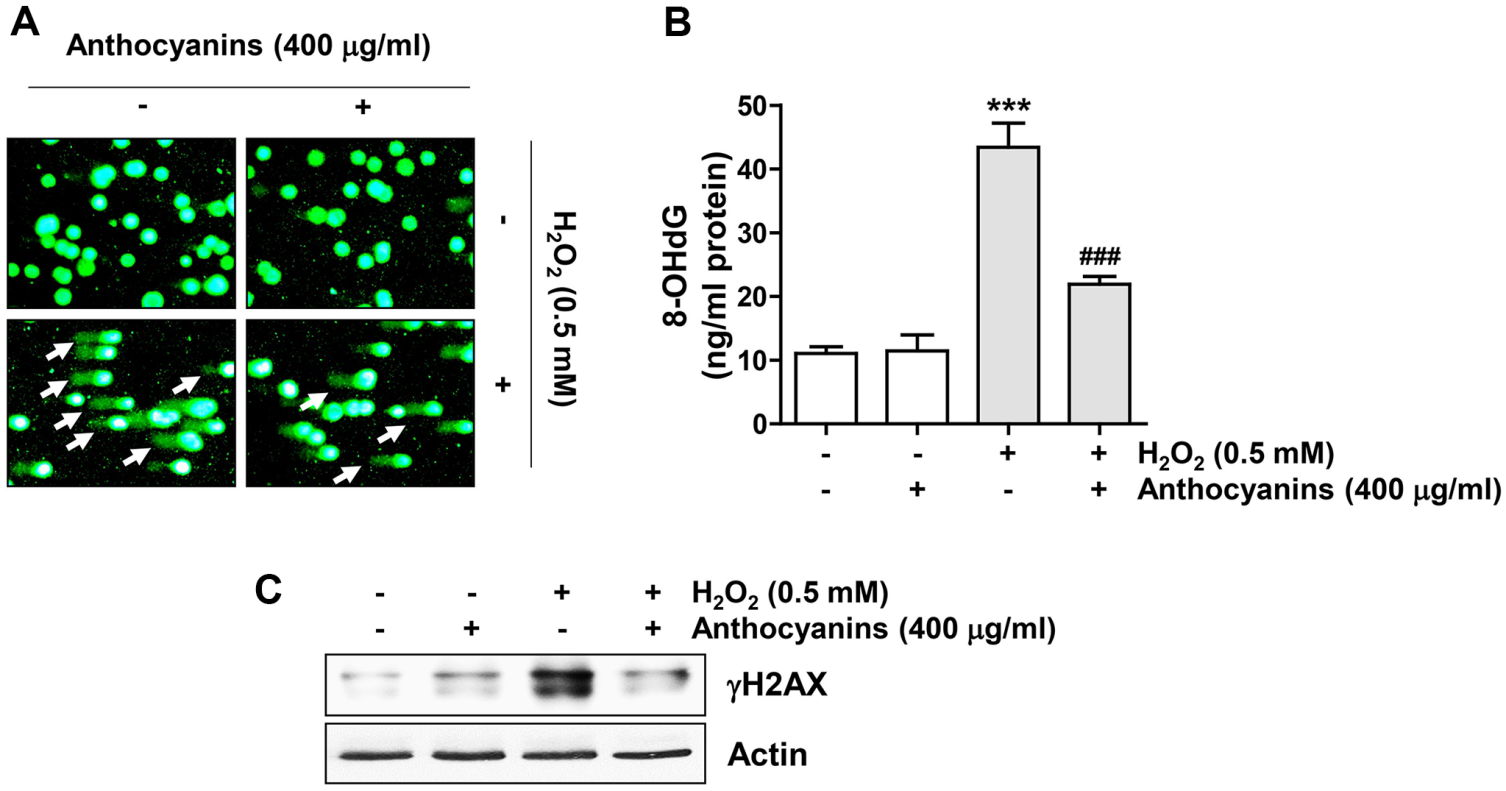
Anthocyanins attenuated DNA damage in H_2_O_2_-treated ARPE-19 cells. Before treating the cells with H_2_O_2_ for 24 h, they were incubated in the presence or absence of anthocyanins for 1 h. Representative images of comet assay (**A**), 8- OHdG levels (**B**) and expression changes of γH2AX (**C**) were presented.

To determine whether the blocking potential of anthocyanins against H_2_O_2_-mediated cytotoxicity is correlated with the prevention of DNA damage, the effects of anthocyanins on comet tail formation, 8-OHdG content and phosphorylation of histone H2AX (γH2AX) by H_2_O_2_ treatment were evaluated. As indicated in Fig. 2, the comet tail movement, 8-OHdG levels and γH2AX expression were greatly increased by H_2_O_2_ treatment, which were markedly weakened by anthocyanins.

### Anthocyanins Inhibited H_2_O_2_-Induced Apoptosis in ARPE-19 Cells

**Fig. 3:**
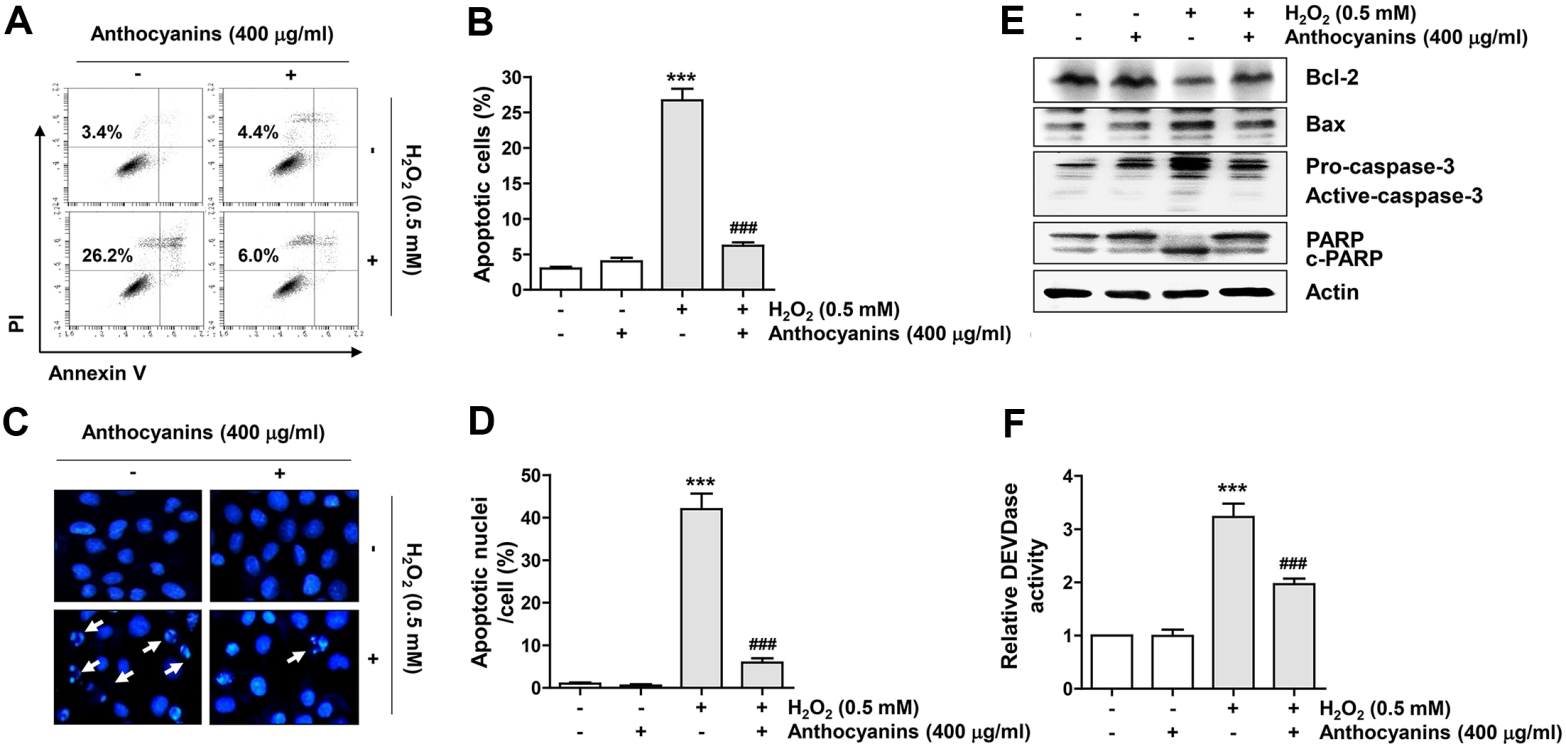
Anthocyanins ameliorated H_2_O_2_-induced apoptosis in ARPE-19 cells. Cells were exposed to anthocyanins for 1 h prior to treatment with H_2_O_2_ for 24 h. (**A** and **B**) Representative histograms (**A**) and quantitative results (**B**) of flow cytometry analysis by Annexin V/PI staining. (**C** and **D**) Images of representative nuclei (**C**, 400×) and results of quantitative analysis obtained after DAPI staining. (**E**) Expression changes of the indicated proteins obtained through immunoblotting. (**F**) Differences in caspase-3 activity by treatment group.

Next, we examined whether anthocyanins affect H_2_O_2_-induced apoptosis. Results from flow cytometry showed that apoptosis was greatly increased by H_2_O_2_ stimulations but was significantly abrogated by anthocyanin pretreatment (Fig. 3A and 3B). Further, in H_2_O_2_-exposed ARPE-19 cells, morphological changes in the nucleus such as nuclear fragmentation and chromatin condensation characteristic of apoptosis were clearly observed. However, these morphological features of apoptosis were significantly attenuated by anthocyanins pretreatment (Fig. 3C and 3D). Moreover, immunoblotting results indicated that H_2_O_2_ treatment suppressed Bcl-2 expression and increased Bax expression, which was related to caspase-3 activation and poly (ADP-ribose) polymerase (PARP) degradation. However, these changes induced by H_2_O_2_ treatment were largely abolished by anthocyanin pretreatment (Fig. 3E and 3F).

### Anthocyanins Reduced Mitochondrial Impairment in H_2_O_2_-Treated ARPE-19 Cells

**Fig. 4:**
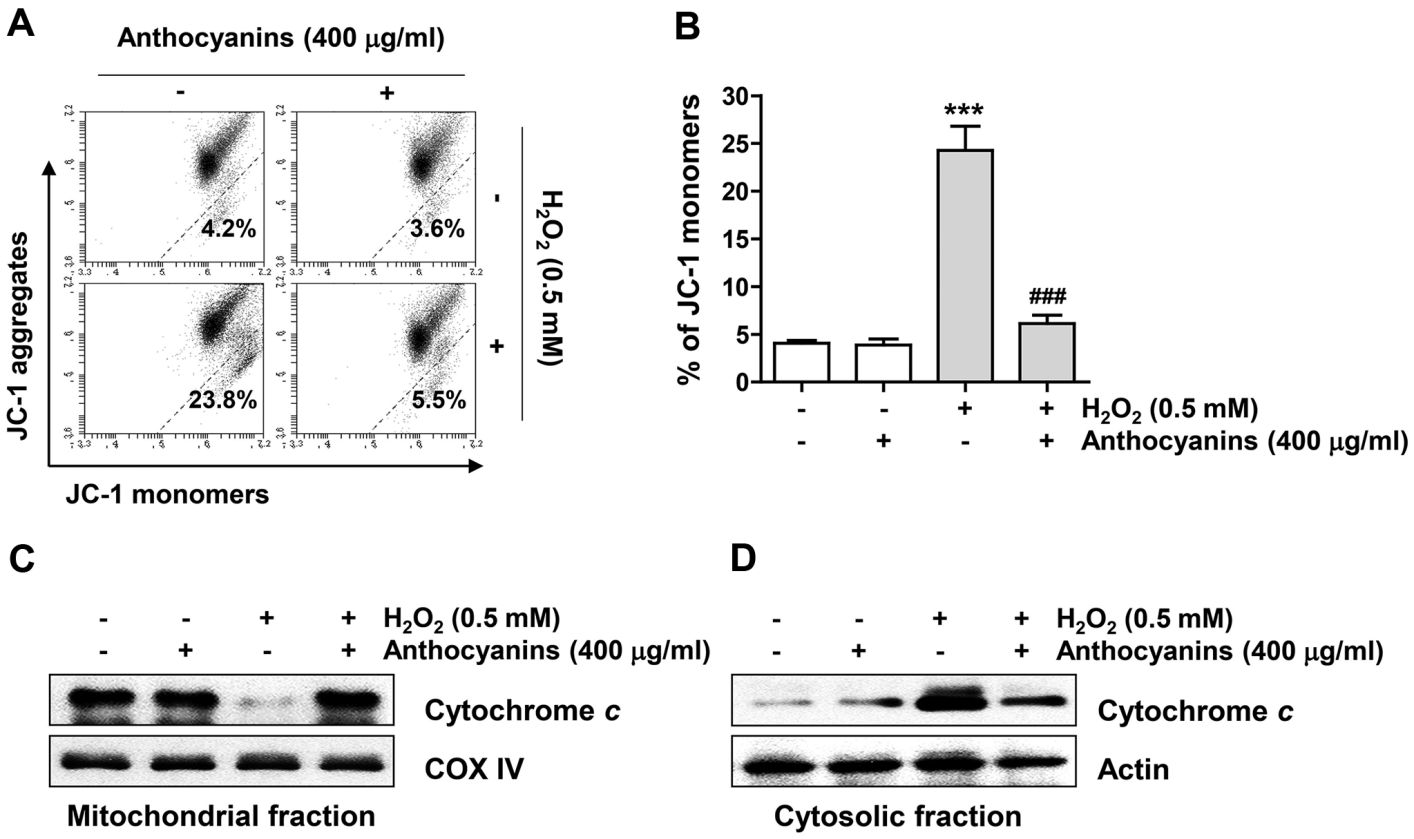
Anthocyanins suppressed H_2_O_2_-induced mitochondrial impairment in ARPE-19 cells. Cells exposed with or without anthocyanins for 1 h were treated with H_2_O_2_ for 24 h. (**A** and **B**) Representative histograms (**A**) and JC-1 monomer ratios (**B**) of flow cytometry using JC-1 staining in each treatment group. (**C** and **D**) The expression of cytochrome c in the mitochondrial and cytosolic fractions was investigated by immunoblotting.

To evaluate whether the suppressive capacity of anthocyanins against H_2_O_2_-mediated apoptosis is associated with their protective ability against mitochondrial damage, MMP was measured. Our data revealed that as much as the frequency of JC-1 monomers increased by H_2_O_2_ treatment, the frequency of JC-1 aggregates was diminished (Fig. 4A and 4B). Moreover, in H_2_O_2_-treated cells, the level of cytochrome c protein was up-regulated in the cytoplasm but down-regulated in the mitochondria (Fig. 4C and 4D). However, anthocyanin mitigated all these changes caused by H_2_O_2_ treatment.

### Anthocyanins Decreased ROS Production and Increased GSH/GSSG Ratio in H_2_O_2_-Exposed ARPE-19 Cells

**Fig. 5:**
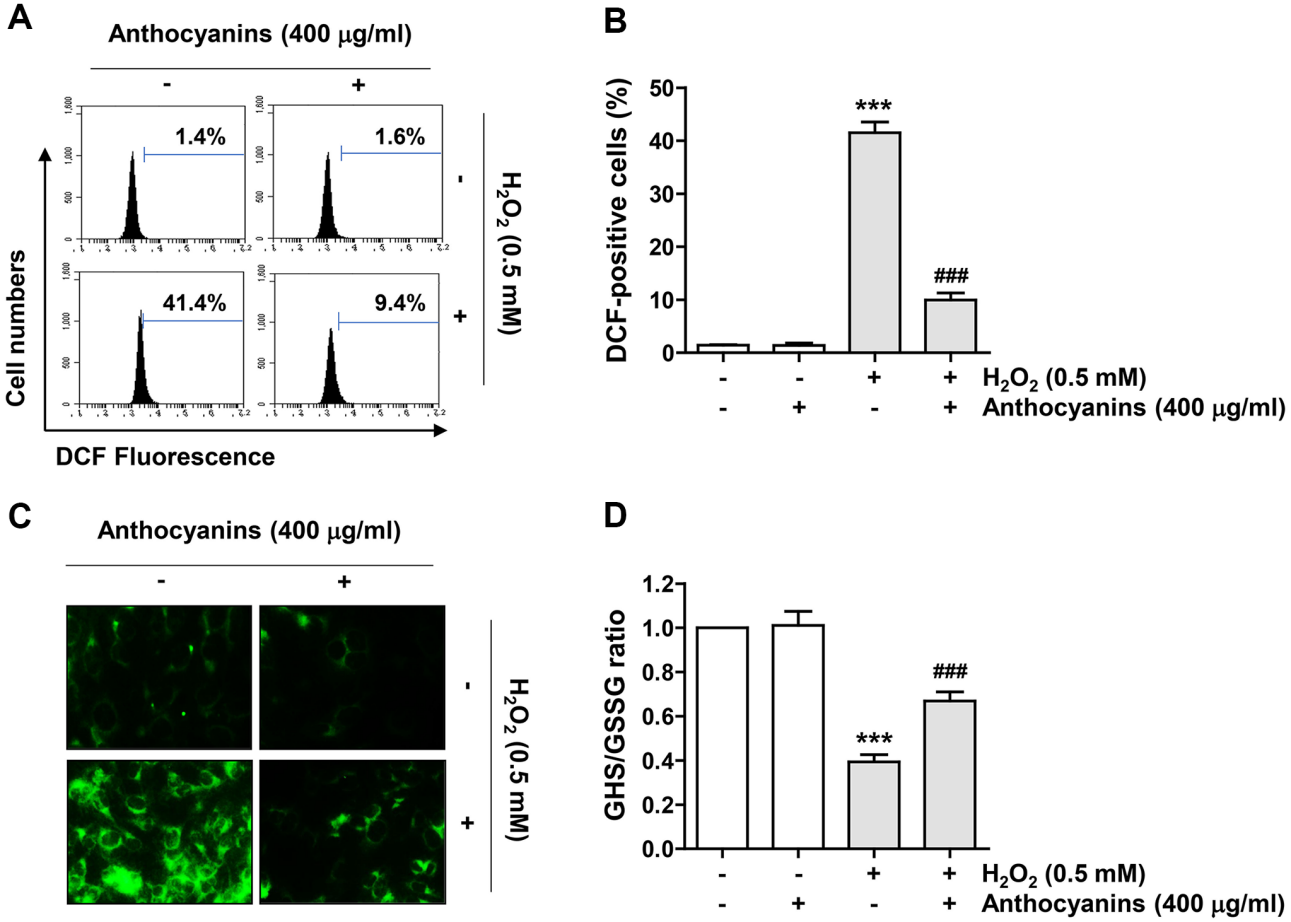
Anthocyanins attenuated ROS production and reduced GSH/GSSG ratio in ARPE-19 cells under H_2_O_2_-treated conditions. Cells exposed with or without anthocyanins for 1 h were stimulated with H_2_O_2_ for 1 h (**A**, **B** and **C**) or 24 h (**D**). (**A** and **B**) Representative histograms of flow cytometry (**A**) and the frequency of DCF-positive cells (**B**). (**C**) Representative fluorescence images of ROS production. (**D**) Bar chart indicated the GSH/GSSG ratio following the exposure to H_2_O_2_ and pretreatment with anthocyanins.

Since mitochondria are the main source of ROS and the primary targets for ROS damage, we investigated the effect of anthocyanins on intracellular ROS formation by H_2_O_2_. Our results showed that the intensity of oxidized DCF, indicating ROS production, was approximately 6-fold higher in cells treated with H_2_O_2_ than in control cells, which was significantly reduced in anthocyanins-pretreated cells (Fig. 5A and 5B). In parallel, fluorescence microscopy revealed strong expression of DCF-fluorescence intensity (green) in H_2_O_2_-treated cells compared to untreated cells (Fig. 5C), which was markedly abrogated by anthocyanins pretreatment. In addition, H_2_O_2_ exposure significantly decreased the GSH/GSSG ratio, but anthocyanins pretreatment significantly increased the reduced GSH level (Fig. 5D).

### Anthocyanins Increased H_2_O_2_-Induced Nrf2 Phosphorylation and HO-1 Activity in ARPE-19 Cells

**Fig. 6:**
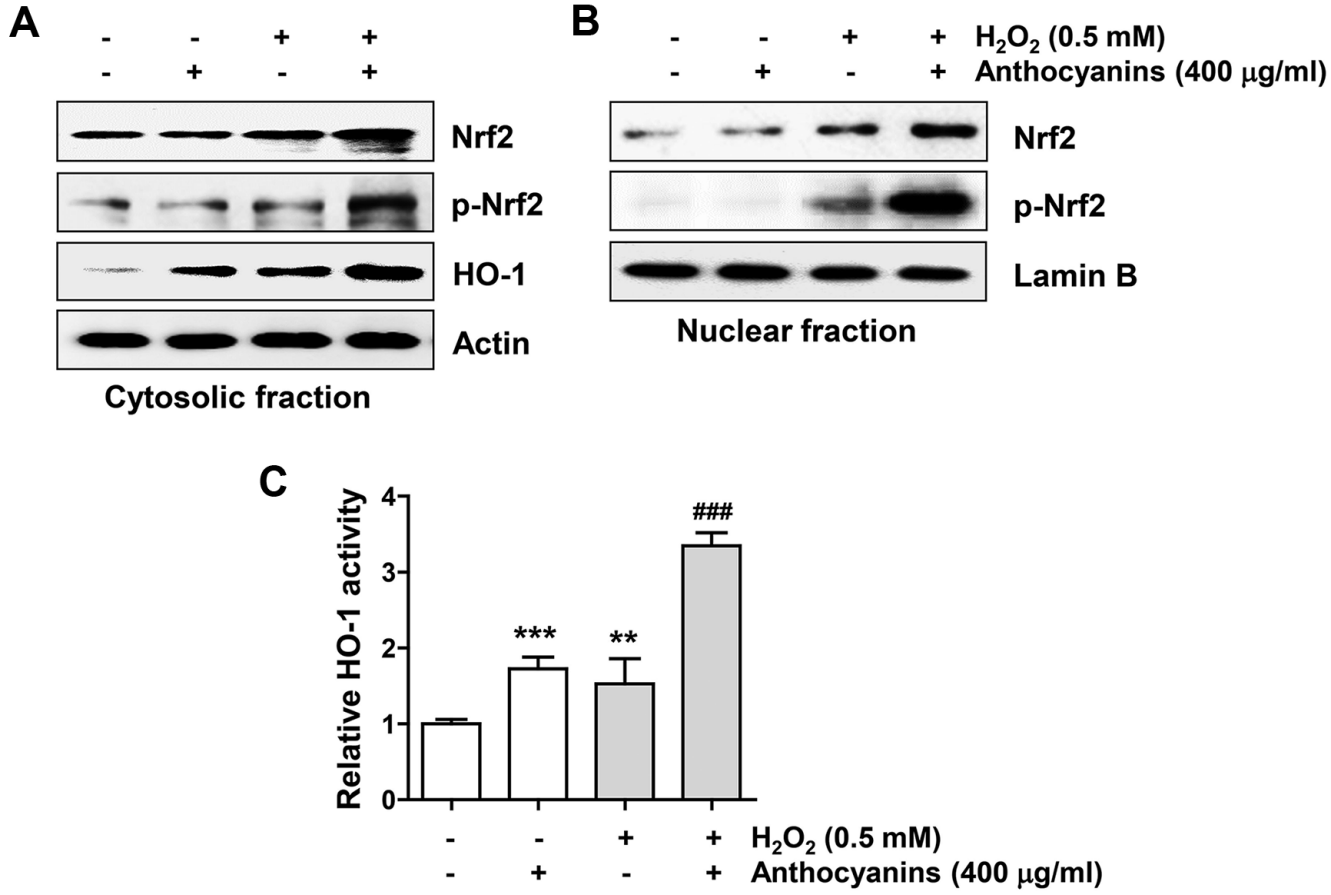
Anthocyanins activated the Nrf2/HO-1 signaling in ARPE-19 cells under H_2_O_2_-treated conditions. Cells exposed with or without anthocyanins for 1 h were stimulated with H_2_O_2_ for 24 h. (**A** and **B**) Expression changes of proteins presented in each treatment group were analyzed by immunoblotting using cytosolic and nuclear fractions. (**C**) The activity of HO-1 in each treatment group was expressed as a relative value.

Next, we examined whether activation of Nrf2, a potent antioxidant transcriptional regulator, was related to the antioxidant capacity of anthocyanins. The data in Fig. 6A and 6B indicate that the total expression of Nrf2 protein and its phosphorylation level (p-Nrf2) were clearly enhanced in cells co-treated with anthocyanins and H_2_O_2_ compared to cells treated with H_2_O_2_ and anthocyanins alone. Furthermore, the activity and expression of HO-1, a key downstream enzyme of Nrf2, were enhanced in cells co-treated with anthocyanins and H_2_O_2_ (Fig. 6A and 6C).

### Role of HO-1 Activation in Inhibition of ROS Production and Recovery of Cytotoxicity by Anthocyanins in ARPE-19 Cells Exposed to H_2_O_2_

**Fig. 7:**
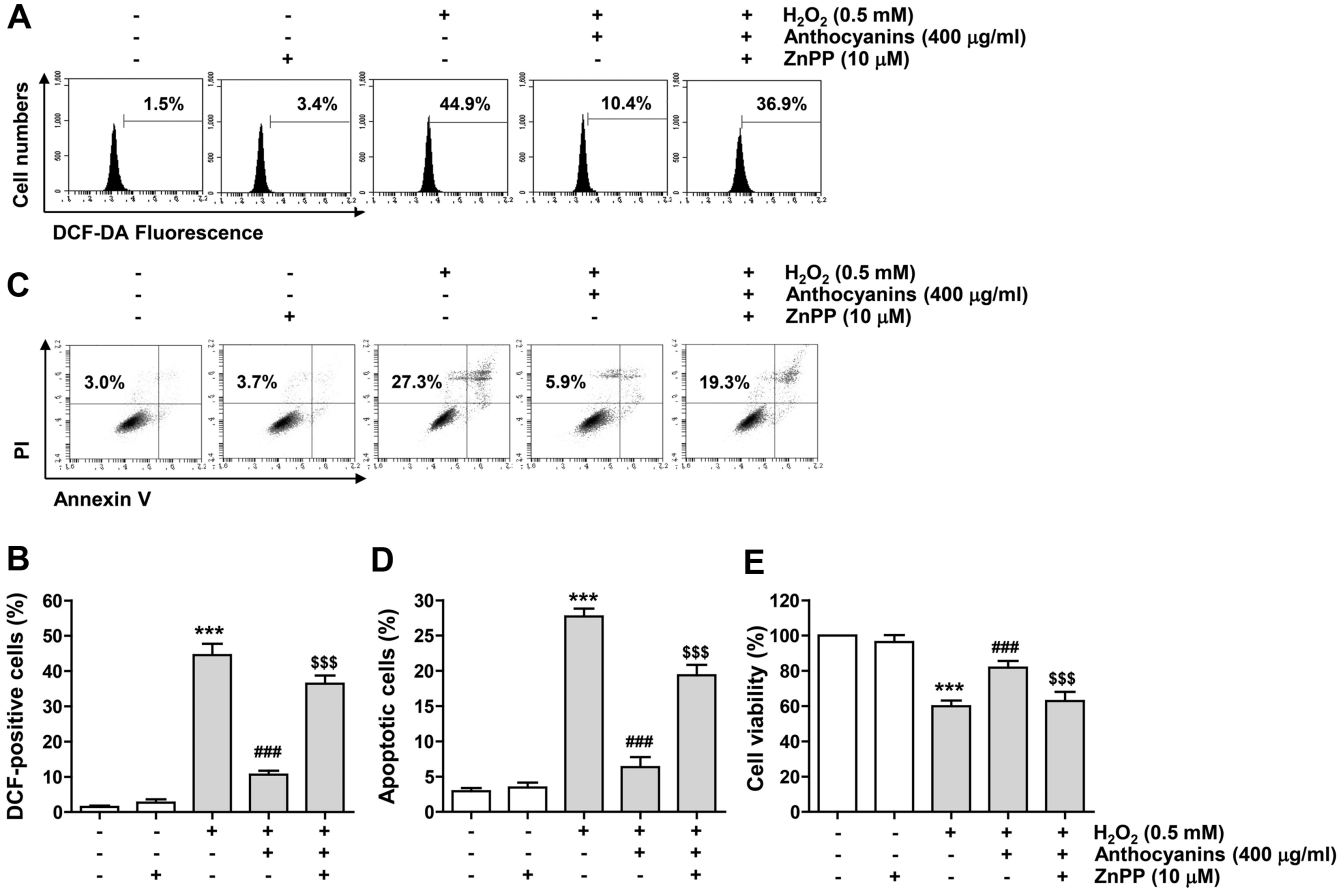
The protective ability of anthocyanins against ROS generation and apoptosis caused by H_2_O_2_ treatment was offset by ZnPP in ARPE-19 cells. Cells were treated with anthocyanins and/or ZnPP for 1 h and then further treated with H_2_O_2_ for 24 h. (**A** and **B**) Representative flow cytometry results (**A**) and their average values (**B**) according to DCF-DA staining. (**C** and **D**) Representative histograms (**C**) and quantitative results (**D**) of flow cytometry analysis following double staining of annexin V and PI. (**E**) Cell viability was assessed using MTT assay.

To investigate whether the increase in HO-1 activity by anthocyanins in ARPE-19 cells exposed to H_2_O_2_ was associated with the antioxidant potential of anthocyanins, we evaluated the efficacy of ZnPP, a competitive blocker of HO-1. As shown in Fig. 7A and 7B, the protective effect of anthocyanins on ROS accumulation caused by H_2_O_2_ was clearly reversed in the presence of ZnPP. And, pretreatment with ZnPP significantly reduced the inhibitory effect of anthocyanins on apoptosis induced by H_2_O_2_ treatment against H_2_O_2_-induced apoptosis (Fig. 7C and 7D). Consistent with these results, ZnPP pretreatment abolished the cytotoxic protective effect of anthocyanins in H_2_O_2_-treated cells (Fig. 7E).

## Discussion

Accumulating studies have demonstrated that DNA and mitochondrial damage induced by oxidative stimuli are closely accompanied by ROS-dependent apoptosis. Previous studies have also shown that the genotoxic effects of H_2_O_2_ on RPE cells are mostly related to mitochondrial dysfunction and apoptosis, which was associated with damage to intracellular macromolecules including DNA [28, 33, 34]. RPE cells exposed to a high oxidative stress environment are susceptible to defense against DNA damage, cellular senescence and apoptosis, and loss of antioxidant capacity underlies degenerative retinal diseases such as age-related macular degeneration [35, 36]. Here, we demonstrated that anthocyanins were able to block DNA damage caused by H_2_O_2_ in ARPE-19 cells, as evidenced inhibiting hallmarks of DNA double-strand breaks, including DNA tail formation and γH2AX expression [37, 38]. Anthocyanins also normalized levels of 8-OHdG, a widely used biomarker of oxidative stress in nucleic acids [39], in H_2_O_2_-treated ARPE-19 cells. Additionally, as analyzed by flow cytometry and DAPI staining, exposure to H_2_O_2_ increased the frequency of apoptosis-induced cells. However, these changes were apparently eliminated after anthocyanins pretreatment.

Since GSH acts as an antioxidant enzyme cofactor and scavenges ROS and electrophiles, the ratio of reduced GSH to oxidized GSSG is used to measure the cellular redox state [40, 41]. Consistent with previous studies on the efficacy of berry-derived anthocyanins reported in ARPE-19 cells irradiated with visible light [42], anthocyanins used in this study significantly reduced H_2_O_2_-induced ROS accumulation while restoring the GSH/GSSG ratio. Excessive ROS due to oxidative stimuli contributes to depolarization of the mitochondrial membrane, resulting in MMP loss, an indicative of mitochondrial impairment [43, 44]. Loss of MMPs in turn triggers the release of mitochondrial cytochrome c into the cytosol, where it activates the caspase cascade, initiating the mitochondria-mediated apoptotic pathway and ultimately cleaving target proteins of effector caspases, including PARP [44, 45]. Similar to previous results [46, 47], in the current study, loss of MMP, cytochrome c release into the cytosol, and degradation of PARP by caspase-3 activation were observed in ARPE-19 cells treated with H_2_O_2_. However, these changes were significantly reduced by anthocyanins pretreatment, and caspase-3 inactivation may be causally related to protection against H_2_O_2_-induced apoptosis.

As is well known, Bcl-2 family proteins are critically involved in the regulation of the apoptosis. Among them, pro-apoptotic proteins such as Bax play a critical role in the formation of mitochondrial pores that disrupt mitochondrial membrane barrier stability, while anti-apoptotic proteins including Bcl-2 play the opposite role [43, 48]. Therefore, when Bcl-2 expression is relatively lower than Bax, mitochondrial membrane permeability increases and mitochondrial cytochrome c release is enhanced [45, 48]. In this study, it was confirmed that the decreased Bcl-2 and increased Bax expression by H_2_O_2_ treatment were restored in the presence of anthocyanins, which may be responsible for the restoration of MMP loss. These results indicate that anthocyanins protected ARPE-19 cells from DNA and mitochondrial damage and induction of apoptosis under conditions of oxidative environment while exerting ROS scavenging activity. Nrf2, a redox-sensitive transcription factor, enhances antioxidant capacity by promoting transcription of phase II detoxification enzymes [18, 19]. Under normal physiological conditions, this transcription factor is located in the cytoplasm bound to its inhibitor, Kelch-like ECH-associated protein 1 (Keap1), and is degraded via the ubiquitin-proteasome pathway. To enhance the transcription of antioxidant genes regulated by Nrf2, Nrf2 must be phosphorylated after dissociation from Keap1 prior to nuclear translocation.

Among the Nrf2-dependent downstream factors, HO-1 break down heme into biliverdin, free iron, and carbon monoxide, of which bilirubin converted from biliverdin exerts strong antioxidant action [19, 20]. Recently, Nrf2-dependent activation of HO-1 in RPE cells was found to contribute to protection against mitochondrial damage-mediated apoptosis caused by oxidative stress [28, 46, 49, 50]. According to the results of this study, anthocyanins increased Nrf2 expression and phosphorylation in H_2_O_2_-treated ARPE-19 cells, and they were expressed predominantly in the nucleus. Furthermore, anthocyanins upregulated HO-1 expression as well as its enzymatic activity, demonstrating that anthocyanins may increase HO-1 expression by acting as activators of Nrf2. In subsequent experiments using the HO-1 inhibitor, the antioxidant potency of anthocyanins to block apoptosis and cytotoxicity in H_2_O_2_-exposed ARPE-19 cells was largely offset, suggesting that HO-1 activation contributed to inhibition of H_2_O_2_-induced oxidative damage by anthocyanins. The current results are similar to previous findings that the antioxidant activity of anthocyanins such as cyanidin-3-glucoside and delphinidin is due to Nrf2-mediated activation of HO-1 in RPE cells [21, 22]. Therefore, the present results indicate that HO-1 activation by anthocyanins contributes at least as one of the upstream signals for the protective action of anthocyanins on H_2_O_2_-mediated cytotoxicity in ARPE-19 cells.

**Fig. 8:**
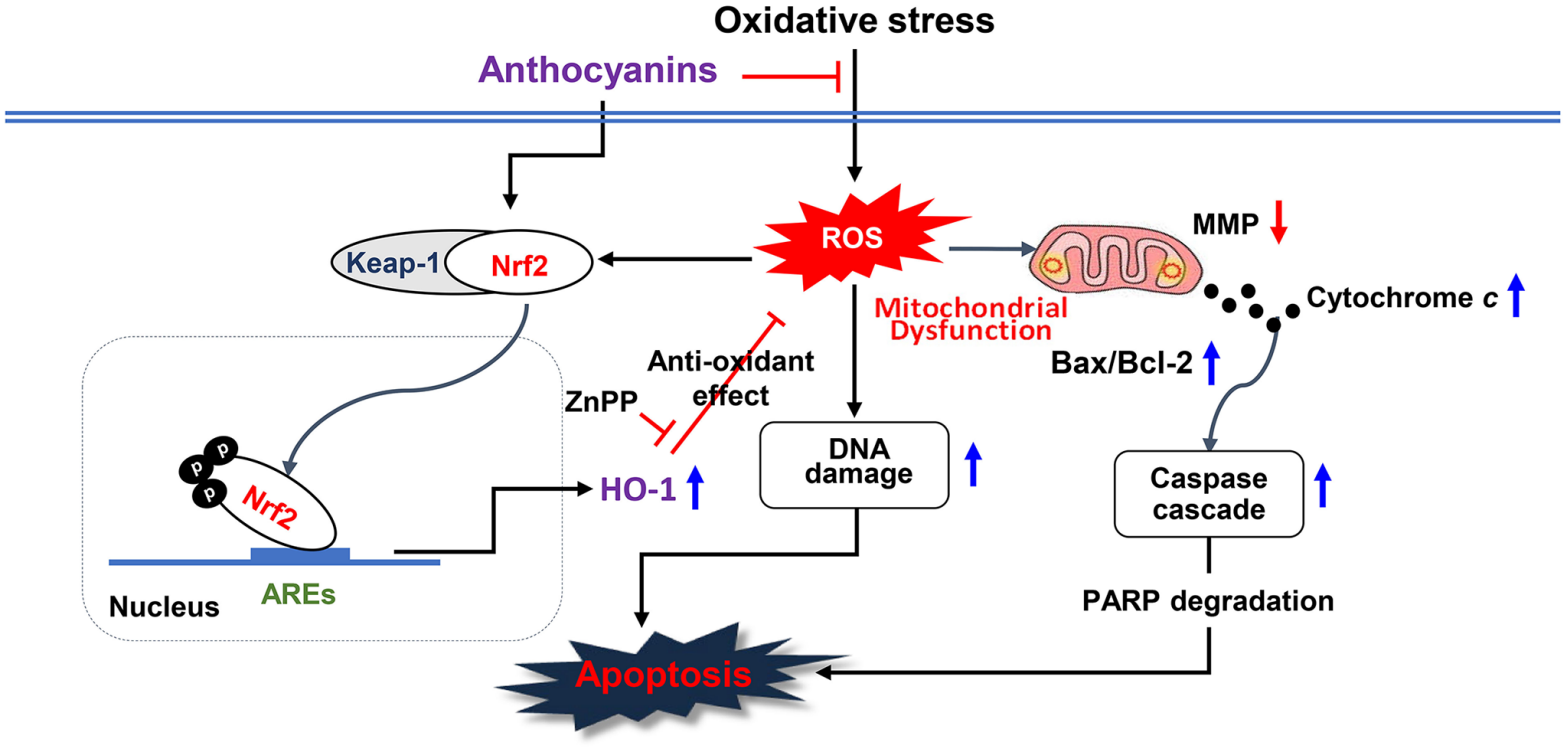
Schematic diagram showing the blocking effect of anthocyanins on oxidative stress in ARPE-19 cells.

Taken together, our data demonstrated that anthocyanins could reduce H_2_O_2_-induced cellular toxicity such as DNA damage and apoptotic cell death by alleviating mitochondrial dysfunction through scavenging ROS and increasing GSH in ARPE-19 cells. Moreover, anthocyanins may contribute to eliminating oxidative stress by enhancing the activation of the Nrf2/HO-1 axis, probably because H_2_O_2_-induced ROS accumulation was suppressed by HO-1 activation (Fig. 8). Although further studies are needed to better understand the mechanisms of upstream regulators regulating Nrf2 phosphorylation, these findings support the preventive potential of anthocyanins in oxidative injury-related ocular disease.

## Figures and Tables

**Table 1:** List of antibodies used in this study.

Antibody	Species raised	Dilution	Product Code	Source
γH2AX	Mouse monoclonal	1:500	MA1-2022	Thermo Fisher Scientific Inc.
Bcl-2	Mouse monoclonal	1:1000	sc-509	Santa Cruz Biotechnology Inc.
Bax	Mouse monoclonal	1:1000	sc-7480	Santa Cruz Biotechnology Inc.
Caspase-3	Rabbit polyclonal	1:1000	#9662	Cell Signaling Technology Inc.
PARP	Mouse monoclonal	1:1000	sc-8007	Santa Cruz Biotechnology Inc.
Cytochrome *c*	Mouse monoclonal	1:1000	sc-13560	Santa Cruz Biotechnology Inc.
Nrf2	Mouse monoclonal	1:1000	sc-518036	Santa Cruz Biotechnology Inc.
p-Nrf2	Rabbit polyclonal	1:500	PA5-67520	Thermo Fisher Scientific Inc.
HO-1	Mouse monoclonal	1:1000	sc-136960	Santa Cruz Biotechnology Inc.
Lamin B	Rabbit polyclonal	1:500	ab65986	Abcam, Inc.
COX IV	Rabbit polyclonal	1:1000	#4844	Cell Signaling Technology Inc.
Actin	Mouse monoclonal	1:1000	sc-47778	Santa Cruz Biotechnology Inc.
